# Optimization of the energy for Breast monochromatic absorption X-ray Computed Tomography

**DOI:** 10.1038/s41598-019-49351-2

**Published:** 2019-09-11

**Authors:** Pasquale Delogu, Vittorio Di Trapani, Luca Brombal, Giovanni Mettivier, Angelo Taibi, Piernicola Oliva

**Affiliations:** 1 Dipartimento di Scienze Fisiche, della Terra e dell’Ambiente, Università di Siena, Siena, Italy; 2 I.N.F.N. Sezione di Pisa, Pisa, Italy; 3 Dipartimento di Fisica, Università di Trieste, Trieste, Italy; 4 I.N.F.N. Sezione di Trieste, Trieste, Italy; 5 Dipartimento di Fisica, Università di Napoli Federico II, Napoli, Italy; 6 I.N.F.N. Sezione di Napoli, Napoli, Italy; 7 Dipartimento di Fisica e Scienze dalla Terra, Università di Ferrara, Ferrara, Italy; 8 I.N.F.N. Sezione di Ferrara, Ferrara, Italy; 9 Dipartimento di Chimica e Farmacia, Università di Sassari, Sassari, Italy; 10 I.N.F.N. Sezione di Cagliari, Cagliari, Italy

**Keywords:** Computed tomography, Applied physics, Biological physics

## Abstract

The limits of mammography have led to an increasing interest on possible alternatives such as the breast Computed Tomography (bCT). The common goal of all X-ray imaging techniques is to achieve the optimal contrast resolution, measured through the Contrast to Noise Ratio (CNR), while minimizing the radiological risks, quantified by the dose. Both dose and CNR depend on the energy and the intensity of the X-rays employed for the specific imaging technique. Some attempts to determine an optimal energy for bCT have suggested the range 22 keV–34 keV, some others instead suggested the range 50 keV–60 keV depending on the parameters considered in the study. Recent experimental works, based on the use of monochromatic radiation and breast specimens, show that energies around 32 keV give better image quality respect to setups based on higher energies. In this paper we report a systematic study aiming at defining the range of energies that maximizes the CNR at fixed dose in bCT. The study evaluates several compositions and diameters of the breast and includes various reconstruction algorithms as well as different dose levels. The results show that a good compromise between CNR and dose is obtained using energies around 28 keV.

## Introduction

At present dual view 2D mammography is the main tool for detecting breast cancer^1,2^. However, this method is mainly limited by the overlap of anatomical structures which can reduce the contrast among breast tissues leading to misinterpretations^3–7^. It has been widely demonstrated that, compared to mammography, breast Computed Tomography (bCT) improves the visibility of the anatomical structures removing the overlaps at the cost of a loss in spatial resolution^8–14^ and increased dose^15–17^. The increasing interest on bCT among radiologists and the need for detecting micro-calcifications have recently led to the development of dedicated breast CT systems with improved spatial resolution and doses comparable to the ones accepted in clinical practice^13,18,19^. Since for planar breast imaging the use of monochromatic synchrotron radiation combined with phase contrast techniques has been demonstrated to improve the diagnostic performance without increasing the dose^20,21^, experimental setups for absorption and phase contrast bCT are being implemented at both the Italian and Australian synchrotron facilities^22,23^. Specifically, the Syrma-3D project, developed at the SYRMEP beamline at Elettra synchrotron (Trieste, Italy), aims to perform the first clinical synchrotron radiation-based bCT^24–32^. In this context, the study hereby presented mimics the Syrma-3D acquisition setup, which is based on the laminar monochromatic synchrotron beam and a photon counting detector with a $650\,\mu m$ thick CdTe sensor and pixel size $\Delta x=0.06\,mm$^33,34^. This particular setup allows to acquire the projections in pure parallel-beam geometry with a complete scan over 180°. Moreover, the height of the beam $h=3.5\,mm$ and the large object-detector distance $ODD=185\,cm$ results in virtually scattering-free projections. A key role in the implementation of systems for medical imaging is played by the calculation of dose, which gives the estimation of radiological risks. Due to its high radiosensitivity, the calculation of dose for breast applications involves only the glandular component of breast tissue, while adipose/fat tissue is considered risk free. Hence, the reference parameter for dose evaluation is the mean glandular dose (MGD), which is defined as the total energy deposited in the glandular tissue divided by the total glandular mass of the breast^35^. For planar mammography with compressed breast, the evaluation of MGD through Monte Carlo (MC) techniques has been reported in several works^36–44^ and makes use of the normalized glandular dose coefficient (DgN), which is a function of the spectrum of the beam, the composition and the size of the compressed breast:

1 $$MGD=K\cdot DgN$$

where *K* is the air kerma at the entrance surface of the irradiated breast. bCT is a 3D imaging method which does not require the breast compression. The adaptation of the methods of MGD estimation even for bCT configuration has been carried out by various authors^8,45–49^. In this work we refer to (Mettivier *et al*.^49^), in which MC calculations of *DgN*_*CT*_ (i.e. the normalized glandular dose coefficient calculated in bCT geometry) have been carried out for an idealized cylindrical breast of homogeneous composition and a laminar monoenergetic beam. In this model, the breast is partially irradiated. Following the notation of the authors we evaluated the dose from:

2 $$MG{D}_{t}=K\cdot Dg{N}_{CT}$$

where *MGD*_*t*_ is the mean glandular dose which includes also the dose delivered to the glandular tissue outside the irradiated volume by the scattered photons. *K* is the air-kerma at the isocenter and, in the parallel-beam geometry, correspond with the entrance air-kerma.

The quality of X-ray images is mainly given by its discriminating power between different details. For large area details, where the spatial resolution of the detector is not an issue, the Contrast to Noise Ratio (CNR) is the fundamental metrics to quantify image quality. For instance, in planar X-ray imaging, to measure the visibility of a detail with respect to its background it can be defined as:

3 $$CN{R}_{planar}=\frac{|{I}_{detail}-{I}_{background}|}{{\sigma }_{background}}$$

where *I*_*detail*_ and *I*_*background*_ are the intensities in large area regions of interest (ROIs) over the detail and the background respectively, and *σ*_*background*_ is the standard deviation in the background ROI. In planar mammography *CNR*_*planar*_ is related to the MGD:

4 $$CN{R}_{planar}\propto \sqrt{MGD}$$

Moreover, at fixed photon fluence, both *CNR*_*planar*_ and MGD are decreasing functions of the energy. In planar mammography the best compromise between image quality and dose is given by the X-ray spectrum that maximizes the ratio CNR^2^/MGD. It has been demonstrated that, depending on the breast size and composition and on the detection system, the optimal energies are in the range 16 keV–27.4 keV^50^.

Various studies on energy optimization in cone-beam bCT have been published in the past^51–53^. In particular Glick *et al*.^51^ developed a parallel cascade model to evaluate the performances of a dedicated bCT imaging system using a CsI flat panel detector in a truncated cone-beam geometry. By using the ideal observer signal-to-noise ratio as figure of merit, the authors concluded that the best response of their system was achieved within the energy range 36 keV–40 keV. Weigel *et al*.^53^ used the metrics of the contrast-to-noise ratio weighted by the square root of the dose (CNRD). Their work is based on numerical simulations and polychromatic experimental data, the geometry of the acquisition is the cone-beam and the composition of the breast is fixed. The authors found that the energy that maximizes CNRD for adipose/glandular tissues depends on the dimension of the breast (diameter) and falls in the range 22 keV–34 keV. Another attempt to estimate the optimal energy was recently performed trough a study using as reference parameter the ratio dose/transmittance^47^. On the basis of such metrics, the authors suggested an optimal energy range from 50 keV up to 60 keV. A recent work on the evaluation of the image quality of monochromatic low dose bCT, based on breast specimens^54^ showed, through the assessment of its quality with objective metrics (signal to noise ratio, spatial resolution and intrinsic quality characteristic)^55^ and subjective radiological scoring by a group of 13 radiologists, that bCT images at 32 keV are more effective than those at 36 keV and 38 keV. This experimental evidence with monochromatic radiation has shown that energies closer to the ones used in planar mammography could be used to improve the diagnostic power of bCT images. However, to our knowledge, at present a complete systematic study to determine the optimal energy range for parallel-beam bCT image quality optimization has not yet been performed. In this work, we evaluated the quality of bCT images through the specific metrics *CNR*_*bCT*_ and studied it as a function of the energy keeping constant *MGD* and varying, as parameters, the diameter *d* of the breast and its composition. The composition is characterized by its “glandularity” *G*, namely the fraction, in weight, of glandular tissue contained in it. The value of *G* can vary in the range $[0,1]$ where $G=0$ corresponds to a totally adipose tissue and $G=1$ to a totally glandular tissue. Assuming a monochromatic parallel-beam, a cylindrical breast, an ideal photon counting detector, the object-detector distance $ODD=185\,cm$ and fixed dose we calculated analytically $CN{R}_{bCT,an}$ for slices reconstructed using the Filtered Back Projection (FBP) algorithm. This particular geometry is very similar to one of the Syrma-3D setup. Moreover, we developed an analytical simulation of the whole process of bCT acquisition and reconstruction. This program allows to use various reconstruction methods, including iterative algorithms for which the process of reconstruction cannot be analytically modeled. Furthermore, the simulation program takes into account the quantum nature of the photons, i.e. their Poissonian statistics. This is realized in the projections by calculating, for each pixel, the expected value and by adding to it a random correction following the Poissonian distribution. Using this program, we produced a set of simulated bCT images on which we measured $CN{R}_{bCT,sim}$ and compared it to $CN{R}_{bCT,an}$. For all the calculations and for the comparison of the analytical model with the simulation, we fixed the dose to $MGD=20\,mGy$. Even though the Syrma-3D final target dose in possible clinical examinations is $MGD < 5\,mGy$, we chose this value to avoid possible misinterpretation in simulations results, due to statistical fluctuations that are dose dependent. However, MGD affects only the absolute value of CNR, but not its energy dependence and the results are still valid at lower doses. For the same reason, in the simulations (and for consistency also in the analytical model) we used as voxel size $\Delta x=0.12\,mm$ (corresponding to a 2 × 2 re-binning of the Syrma-3D detector). Finally, using the simulation, we studied the visibility of the details at lower doses when the probability of having no counts in some pixels of the detector becomes not negligible. This particular condition can produce the so-called photon starvation artifacts that can further limit the visibility of the details in bCT^56^.

## Methods

### General assumptions

We assume the following hypothesis:

- monochromatic parallel-beam
- ideal cylindrical breast sample of different diameters and compositions
- ideal photon counting detector (the output of the detector reproduces its input)
- absorption imaging
- object-detector distance $ODD=185\,cm$ filled with air
- scattered photons do not contribute to the images

The main components of the breast are the adipose tissue and the glandular tissue. These tissues are characterized, in monochromatic X-ray imaging, by the linear attenuation coefficients ${\mu }_{Adipose}(E)$ and ${\mu }_{Glandular}(E)$ depending only on the energy *E*. In our study we model the breast as a cylindrical phantom of diameter *d*. This phantom contains a homogeneous material specified by its glandularity *G* (i.e. the glandular weight fraction). The attenuation coefficients of pure adipose, glandular and mixed materials (homogeneous materials with specified glandularity *G*) have been determined from their elemental compositions and densities reported in (Hammerstein *et al*.^35^ and Boone and Chavez^57^). In order to take into account inter-individual variability in the female population, we evaluated different values of breast size *d* and composition *G*:

- $$d=\{8\,cm;10\,cm;12\,cm;14\,cm;16\,cm\}$$
- $$G=\{0;0.143;0.25;0.5;0.75;1\}$$

In particular, the value $G=0.143$ was investigated in order to mimic a realistic average glandularity, according to (Yaffe *et al*.^58^).

In our model we supposed to acquire a bCT scan of the ideal phantom, placed with the rotation center in the axis of the cylinder, using a wide monochromatic parallel-beam. The parameters that can be varied are:

- *d* = diameter of the phantom;
- *G* = glandularity of the phantom;
- *MGD* = Mean Glandular Dose;
- *E* = Energy of the photons;
- Δ*x* = pixel size of the detector.

Fixing Δ*x*, for a given set of *MGD*, *E*, *d* and *G* and using the Eq. 2, we calculated *K*. The photon fluence $\varphi$ is obtained from the expression of the air kerma at low energies^59^:

5 $$K={(\frac{{\mu }_{en}}{\rho })}_{air}\cdot E\cdot \varphi $$

In Eq. 5, ${(\tfrac{{\mu }_{en}}{\rho })}_{air}$is the mass energy-absorption coefficient for air^60^. Finally we calculated the total number *N*_*ph*_ of photons per pixel impinging on the phantom by multiplying the fluence and the pixel area:

6 $${N}_{ph}={N}_{ph}(d,G,MGD,\Delta x,E).$$

### Analytical model

In the analytical model, we define the Contrast to Noise Ratio in bCT ($CN{R}_{bCT,an}$) as follows:

7 $$CN{R}_{bCT,an}=\frac{{\mu }_{Glandular}(E)-{\mu }_{Adipose}(E)}{{\sigma }_{center}(d,G,MGD,\Delta x,E)}$$

where ${\mu }_{Adipose}(E)$ and ${\mu }_{Glandular}(E)$ are the linear attenuation coefficients of the adipose and glandular tissues. Equation 7 was chosen in order to mimic CNR measurement in experimental images where the contrast is usually evaluated between bright (typically glandular) and dark (typically adipose) regions. The fluctuations depend instead on the actual mean breast composition and thickness. For the calculation of the noise, in the analytical model the phantom was supposed to be totally homogeneous. Its linear attenuation coefficient ${\mu }_{Phantom}(G,E)$, depends on *G*. If the Filtered Back-Projection with cubic voxel of linear size Δ*x* is used to reconstruct the bCT images, the noise in the center of the reconstructed slice can be expressed by its standard deviation:

8 $${\sigma }_{center}(d,G,MGD,\Delta x,E)=\sqrt{\tfrac{{\rm{\beta }}}{{N}_{ph}(d,G,MGD,\Delta x,E)\cdot {e}^{-{\mu }_{Phantom}(G,E)\cdot d}\cdot {e}^{-{\mu }_{air}(E)\cdot ODD}}}$$

The Eq. 8 was derived, as shown in the Additional Materials, by integrating the formula of the Noise Power Spectrum (NPS) for CT images^61,62^. In the calculation, the parameter *β* depends on the used filter, the pixel size and the interpolation kernel of the reconstruction algorithm. For a pixel size of 0.12 *mm* and a linear interpolation kernel, β_*ramlak*_ = $26.49\,m{m}^{-2}$ for Ram-Lak filter and β_*Hamming*_ = $4.41\,m{m}^{-2}$ for Hamming filter. A detailed derivation *β* is provided in the Additional Materials. Fixing the dose $MGD=20\,mGy$ and the voxel size $\Delta x=0.12\,mm$, we calculated, for a given pair $(d,G)$, the total number of photons per pixel *N*_*ph*_ for each energy in the range $E=[10\,keV,50\,keV]$ with steps of 1 *keV*. This allows us to plot $CN{R}_{bCT,an}$ as a function of the energy, to study its behavior and to calculate its maximum value and the corresponding energy. We repeated this procedure for all the possible $(d,G)$ pairs under investigation, in order to study the energy dependence of the maximum of $CN{R}_{bCT,an}$ for various diameters and glandularities. We compared also the dependence from $(d,G)$ of the energy that maximizes $CN{R}_{bCT,an}$ normalizing the curves to its maximum.

### Simulation program

**Figure 1:**
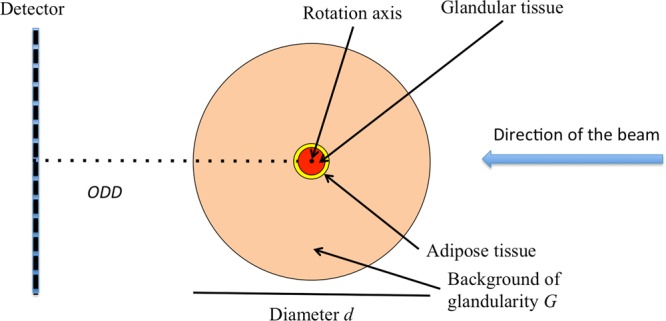
Sketch of the ideal breast used in the simulation program of this study.

We developed a MATLAB (Release 2017b, The MathWorks, Inc., Natick, Massachusetts, United States) program, to analytically simulate the whole process of bCT acquisition and reconstruction. The breast is here modeled as a cylindrical phantom of diameter *d* and glandularity *G*, namely containing a homogeneous material specified by its ${\mu }_{Phantom}(G,E)$. We focused on a slice having a central circular detail of 1 *cm* diameter made of glandular tissue with ${\mu }_{Glandular}(E)$, surrounded by a ring of the same area, made of adipose tissue with ${\mu }_{Adipose}(E)$. A sketch of such phantom is reported in Fig. 1.

To simulate the CT scan acquisition, we fixed the value of *MGD*, Δ*x* and the number of projections *n*_*proj*_ per scan. Then, for a given triplet $(d,G,E)$, we calculated the expected number of input photons per pixel per projection (namely *N*_*ph*_/*n*_*proj*_), simulated the forward projection and generated the sinogram of the slice. In this phase we took into account the quantum nature of the photons and their Poisson distribution and assumed the detector to be an ideal photon counter. It should be noted that this implies that a simulation run gives a single realization of a random process. The reconstruction, based on ASTRA (All Scales Tomographic Reconstruction Antwerp) toolbox^63^, can be made by using all the available algorithms in this environment, for instance the FBP with different filters as well as various iterative methods.

To quantify the quality of the reconstructed slice we used the following metrics:

9 $$CN{R}_{bCT,sim}=\frac{{I}_{Detail}-{I}_{Ring}}{{\sigma }_{Ring}}$$

where *I*_*Detail*_ and *I*_*Ring*_ are the measured mean values in the central detail and the surrounding ring and *σ*_*Ring*_ is the measured standard deviation in the ring. Using the simulation program, we fixed ${n}_{proj}=1200$ over 180° and studied:

- all the available (*d*, *G*) pairs;
- the energies in the range $E=[10\,keV,50\,keV]$ with steps of 1 *keV*;
- FBP reconstruction with Ram-Lak and Hamming filters;
- iterative reconstruction with SART (1200 iterations) and SIRT (500 iterations);
- different values of *MGD* to study photon starvation effects.

We compared the behavior and the absolute value of $CN{R}_{bCT,sim}$ with respect to $CN{R}_{bCT,an}$. We also analyzed the effect of the reconstruction algorithm on the absolute value on $CN{R}_{bCT,sim}$ and on its dependence from the energy. Finally, we studied the effect of decreasing *MGD* down to 1 mGy. If all the other parameters are fixed, we can write:

10 $$CN{R}_{bCT,an}\propto \sqrt{MGD}$$

A similar expression is valid also for $CN{R}_{bCT,sim}$ except in case of very low *MGD* when the ratio *N*_*ph*_/*n*_*proj*_ is small and the probability of having no counts in some pixels of the detector becomes not negligible.

## Results

**Figure 2:**
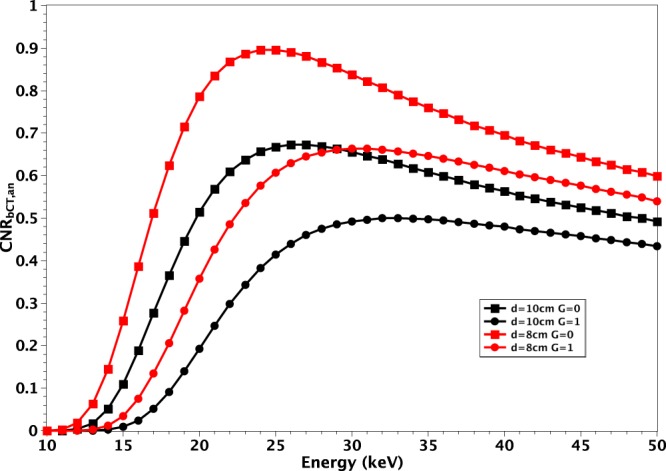
Plot of $CN{R}_{bCT,an}$ for $d=8\,cm$ (red) and $d=10\,cm$ (black) with $G=0$ (squares) and $G=1$ (circles).

For a given pair (*d*, *G*), the plot of $CN{R}_{bCT,an}$ always shows a maximum inside the energy range under investigation. The shape of $CN{R}_{bCT,an}$, the position and the value of its maximum depend on the values of *d* and *G* as exemplified in Fig. 2 where the FBP with Ram-Lak filter was considered.

In particular, when *d* or *G* increases, the value of $CN{R}_{bCT,an}$ decreases at all the energies. In addition, the energy corresponding to the maximum shifts towards higher values. Finally, the curves flatten. Referring to Fig. 2, the maximum value of $CN{R}_{bCT,an}$ for a breast with $d=8\,cm$ and $G=0$ is 1.42 at 23 *keV*. In the case of same composition and $d=10\,cm$, the maximum of $CN{R}_{bCT,an}$ decreases to 1.06 and the corresponding energy shifts to 25 *keV*. Finally, in the case $d=8\,cm$ and $G=1$, the maximum of $CN{R}_{bCT,an}$ decreases to 0.97 and the corresponding energy shifts to 30 *keV*. The values of the maximum of $CN{R}_{bCT,an}$, for FBP with Ram-Lak filter and the corresponding energies are reported in Table 1 for all the investigated (*d*, *G*) pairs.

**Table 1:** Maxima of *CNR*_*bCT*,*an*_ and corresponding energies for all the (*d*, *G*) pairs under investigation.

*d*\*G*	*G* = 0	*G* = 0.143	*G* = 0.25	*G* = 0.5	*G* = 0.75	*G* = 1
*d* = 8 *cm*	1.42 @ 23 *keV*	1.34 @ 24 *keV*	1.28 @ 25 *keV*	1.17 @ 27 *keV*	1.07 @ 28 *keV*	0.97 @ 30 *keV*
*d* = 10 *cm*	1.06 @ 25 *keV*	0.98 @ 27 *keV*	0.95 @ 27 *keV*	0.87 @ 29 *keV*	0.79 @ 31 *keV*	0.73 @ 32 *keV*
*d* = 12 *cm*	0.81 @ 27 *keV*	0.75 @ 29 *keV*	0.72 @ 29 *keV*	0.66 @ 32 *keV*	0.60 @ 34 *keV*	0.56 @ 36 *keV*
*d* = 14 *cm*	0.62 @ 29 *keV*	0.59 @ 31 *keV*	0.56 @ 32 *keV*	0.51 @ 34 *keV*	0.47 @ 36 *keV*	0.44 @ 40 *keV*
*d* = 16 *cm*	0.48 @ 32 *keV*	0.45 @ 32 *keV*	0.44 @ 34 *keV*	0.39 @ 37 *keV*	0.37 @ 40 *keV*	0.34 @ 42 *keV*

FBP with Ram-Lak filter was used to obtain the values.

**Figure 3:**
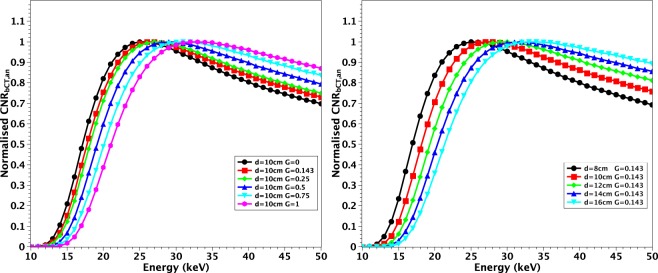
Plots of normalized $CN{R}_{bCT,an}$ for $d=10\,cm$ and various *G* (left) and for $G=0.143$ and various *d* (right).

Even if differences in the optimal energy can be observed, it is important to note that, especially in case of large values of *𝑑* and *𝐺*, the values of $CN{R}_{bCT,an}$ are very close in a broad interval of energies around the maximum. For example, for $d=14\,cm$, $G=0.5$ and FBP with Ram-Lak filter, $CN{R}_{bCT,an,MAX}=0.51$ at 34 keV and $CN{R}_{bCT,an} > 0.44$ in the broad interval $E=[27\,keV,50\,keV]$. The shift in energy and the flattening of the curves are displayed also in Fig. 3 where the plots are normalized to their maxima.

**Figure 4:**
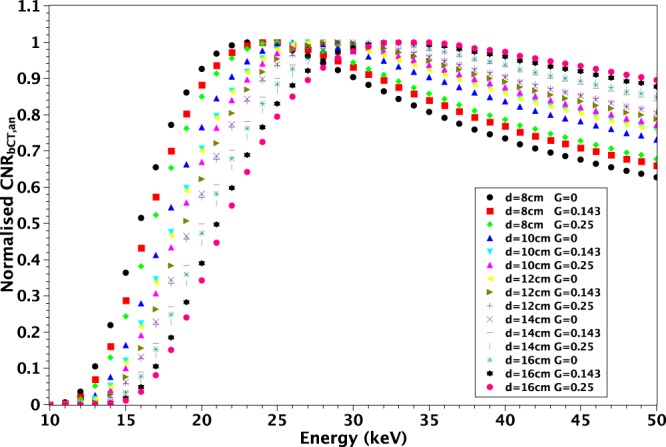
Plots of normalized $CN{R}_{bCT,an}$ for $d=[8\,cm,16\,cm]$ and various $G=[0,0.25]$.

Figure 3 suggests that it is possible to identify a single energy where all the curves are close to each other and $CN{R}_{bCT,an}$ values are near to their maximum. At constant $d=10\,cm$, for all possible *G*, the values $CN{R}_{bCT,an}\,$ are all above the 97% of the maxima at $E=29\,keV$. Likewise fixing $G=0.143$, the values $CN{R}_{bCT,an}$ are all above the 96% of the maxima for all $d=[8\,cm,16\,cm]$ again at $E=29\,keV$. In Fig. 4 we show a global comparison in which all the investigated *d* values are included and the glandularity is limited to the range $G=[0,0.25]$. This assumption excludes the cases of breasts of very highly glandularity that, according to (Huang *et al*.^64^), are highly rare in female population. In this case, at $E=28\,keV$, the value of $CN{R}_{bCT,an}$ is still greater than 93% of its maximum for all the investigated configurations.

**Figure 5:**
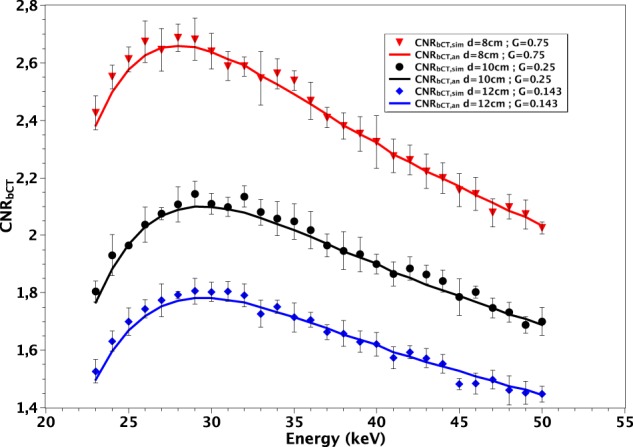
Comparison between $CN{R}_{bCT,an}$ (continuous lines) and $CN{R}_{bCT,sim}$ (symbols) for various *d* and *G*. The used reconstruction algorithm is FBP with Hamming filter. The error bars represent the standard deviation in the $CN{R}_{bCT,sim}$ values measured over 5 different realizations.

In Fig. 5 we compare the $CN{R}_{bCT,an}$ with $CN{R}_{bCT,sim}$ for some pairs (*d*, *G*). The used reconstruction algorithm is FBP with Hamming filter. For each energy, the simulation program was ran 5 times in order to evaluate the errors in measuring $CN{R}_{bCT,sim}$ over multiple realizations. The matching between $CN{R}_{bCT,an}$ and $CN{R}_{bCT,sim}$ is very good both in terms of energy dependence and absolute CNR values.

**Figure 6:**
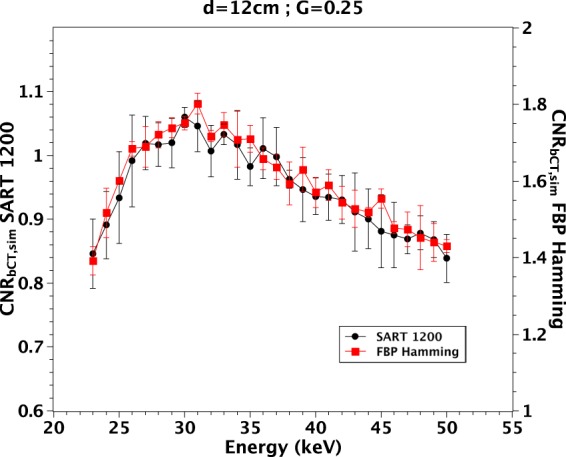
$CN{R}_{bCT,sim}$ measured on a phantom with $d=12\,cm$ and $G=0.25$ with 2 different reconstruction algorithms. The error bars represent the standard deviation in the $CN{R}_{bCT,sim}$ values measured over 5 different realizations.

It is worth noting that the different reconstruction algorithms we have tested act only on noise, thus modifying the absolute value of CNR without changing its shape against the energy. Hence the previous results about the optimal energies, even though obtained supposing FBP, apply also to any of the other tested reconstruction algorithm. This is confirmed by Fig. 6 where the curves of $CN{R}_{bCT,sim}$ ($d=12\,cm$, $G=0.25$) for FBP with Hamming and SART iterative algorithm (1200 iterations) are compared.

**Figure 7:**
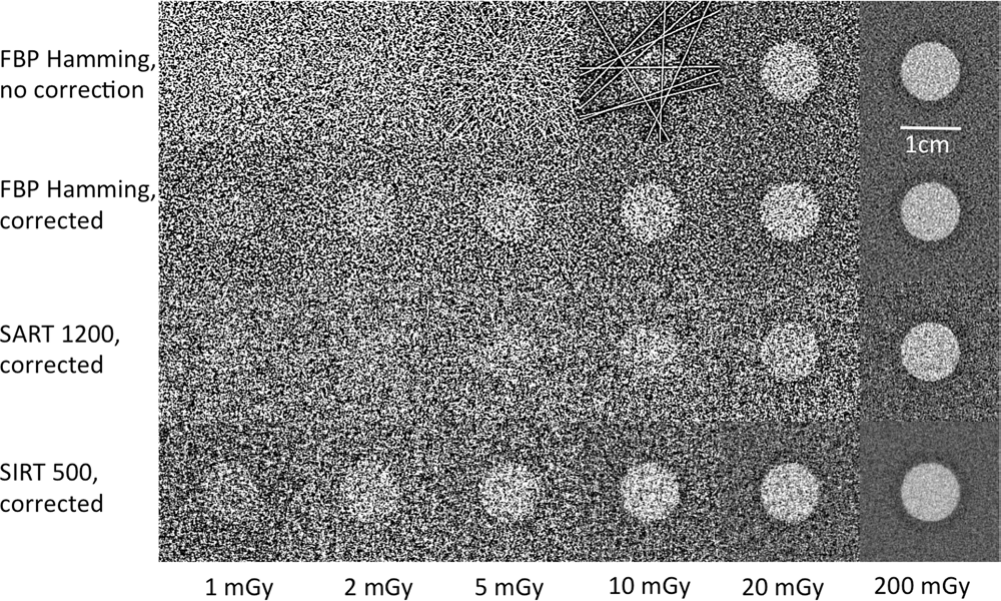
Detail of the reconstructions of simulated bCT of the phantom with $d=16\,cm$, $G=0.143$ and $E=28\,keV$ at different values of *MGD*. In the rows 2, 3 and 4 the photon starvation has been corrected by masking the pixels with zero counts.

In Fig. 7 we show the reconstruction of the central detail and the surrounding ring for the phantom with $$d=16\,cm$$ and $$G=0.143$$. The energy was $$E=28\,keV$$ and the values of *MGD* were: $$\{1\,mGy;2\,mGy;5\,mGy;$$$$10\,mGy;20\,mGy;200\,mGy\}$$. The photon starvation effect appears in the case $$MGD=10\,mGy$$ where 9 different pixels at different projections had no counts, thus originating the streaking artifact visible in the first row of Fig. 7. Decreasing the radiation dose, e.g., $$MGD=5\,mGy$$, the artifact becomes so strong that the central detail cannot be distinguished. However, the effect can be well corrected by masking the pixels with zero counts, via linear interpolation, as shown in the other rows of Fig. 6. This allows obtaining images at very low *MGD*, where the quality is mainly limited by the low value of $$CN{R}_{bCT,sim}$$ and not by the photon starvation artifacts. Moreover, the photon starvation can be simply avoided by limiting the number of projections or by increasing the voxel size, obviously at the cost of the deterioration of the spatial resolution.

## Discussion

Our study demonstrates that, at fixed *MGD*, for a breast of diameter *d* and glandularity *G* a specific energy that maximizes the $CN{R}_{bCT,an}$ exists. This energy is generally lower than the one recommended by other previous studies^47,54^ and is in agreement with the value obtained by (Weigel *et al*.^53^) in the case of $G=0.5$. The results obtained with the analytical model have been confirmed by the simulation software. We showed that the reconstruction algorithm strongly influences the absolute value of $CN{R}_{bCT,sim}$ but does not modify its energy dependence. Similar observations can be made regarding any processing of acquired data that acts independently from the energy. In conclusion, at fixed *MGD*, the energy dependence of $CN{R}_{bCT}$ is defined only by the values of *d* and *G* and a single energy that maximizes it exists. Of course, regardless of the selected energy, an increase of the absolute value of $CN{R}_{bCT}$ can be obtained either by varying *MGD* or by changing the reconstruction algorithm (for example including a smoothing filter) and/or the voxel size, at the cost of a deterioration of the spatial resolution. A decrease in *MGD* causes a reduction in the image quality mainly due to the drop of $CN{R}_{bCT}$ since the photon starvation artifacts represent a less critical issue. Limiting the analysis to breasts with diameters in the range $d=[8\,cm,16\,cm]$ and glandularity in the range $G=[0,0.25]$ and choosing the energy of the photons $E=28\,keV$, we found that the value of $CN{R}_{bCT,an}$ is still greater than 93% of its maximum as shown in Fig. 4. This suggests that this energy can be considered appropriate in most cases.

We underline that the effect of a real detector could be significant on the results especially if its response (in terms of efficiency, spatial resolution and noise) is energy dependent. In this case, the energy response of the detector has to be taken into account and the optimal energies may be different. However, our assumption of an ideal detector is rather realistic for photon-counting devices based on thick CdTe sensors, for the energy range used in this study^33^. Different results can be obtained also when considering different acquisition geometries. For instance, a variation in the *ODD* can cause a shift in the optimal energy. Indeed we expect that, whereas air attenuation is stronger for lower energies, shorter *ODD* values would shift the optimal energies to lower values. Moreover, when drastically reducing *ODD*, for example in a geometry with the detector close to the sample, or in the case of a much wider X-ray beam, the photon scattering cannot be neglected. Since the scattered fraction of interacting photons grows when increasing the energy, we expect a general reduction of the absolute value of the *CNR*_*bCT*_ and a further shift to lower energies when taking into account the scattering. The amount of such energy shift can be quantified only performing specific calculations for the actual geometry.

Finally, we want to underline that our methods have been developed to mimic the imaging at a specific synchrotron beamline and hence a direct, quantitative comparison with bCT systems^52,53^ based on cone-beam geometry, polychromatic beams and detectors with an energy dependent response is not possible at present. However, we believe that our methods to calculate the optimal energy in monochromatic, parallel-beam bCT can pave the way to future studies of more complex cases of systems based on polychromatic and divergent beams and detectors with an energy and intensity dependent response.

## Supplementary information

Additional Materials
