# Supplementary material for: Optimization of the energy for Breast monochromatic absorption X-ray Computed Tomography
Source: Sci Rep. 2019 Sep 11;9:13135. doi: 10.1038/s41598-019-49351-2 (PMC6739417; doi:10.1038/s41598-019-49351-2)
Supplement: Supplementary file 1 — Additional Materials [file 41598_2019_49351_MOESM1_ESM.pdf]

Optimization of the energy for Breast monochromatic absorption X-ray Computed Tomography

Pasquale Delogu[a*], Vittorio Di Trapani[a], Luca Brombal[b], Giovanni Mettivier[c], Angelo Taibi[d], Piernicola Oliva[e]

[a] *Dipartimento di Scienze Fisiche, della Terra e dell'Ambiente, Università di Siena and I.N.F.N. Sezione di Pisa.*

[b] *Dipartimento di Fisica, Università di Trieste and I.N.F.N. Sezione di Trieste.*

[c] *Dipartimento di Fisica, Università di Napoli Federico II and I.N.F.N. Sezione di Napoli.*

[d] *Dipartimento di Fisica e Scienze dalla Terra, Università di Ferrara and I.N.F.N. Sezione di Ferrara.*

[e] *Dipartimento di Chimica e Farmacia, Università di Sassari and I.N.F.N. Sezione di Cagliari.*

Additional Materials

In spatial domain, the noise of CT images is defined as the standard deviation σ measured on a homogeneous ROI (Region Of Interest). In frequency domain, the Noise Power Spectrum (NPS) describes the component of the variance σ^2 for each spatial frequency. The variance σ^2 can be calculated integrating the 2D NPS over the frequency domain [1]

$$\sigma^2 = \int_{-\infty}^{\infty} \int_{-\infty}^{\infty} NPS(f_x, f_y)\, df_x\, df_y \qquad (1)$$

For CT reconstructions with FBP algorithm, the theoretical NPS has been previously reported by various authors. [2,3,4,5,6]

As reported by (Riederer *et al* 1978[2]), the NPS for a single reconstructed voxel can be written as follows:

$$NPS(q) = \frac{\pi \Delta x}{m\langle n\rangle} \cdot \frac{|W(q)|^2}{q} \qquad (2)$$

where $q = \sqrt{x^2 + y^2}$ is the spatial frequency in polar coordinates, $\langle n\rangle$ the average number of photons per pixel per projection, m the number of projections, Δx the pixel size and |W(q)| the spectral response of the reconstruction algorithm. In particular (Kijewski and Judi 1987 [6]), considering also the linear interpolations used by the reconstruction algorithm to estimate the projection values between the measured points during the backprojection, derived the following mathematical formulation for $|W(q)|^2$ in FBP algorithm:

$$|W(q)|^2 = \left(sinc^2(\pi q \Delta x)\right)^2 \cdot \sum_{k=-\infty}^{+\infty} \left(q - \frac{k}{\Delta x}\right)^2 \cdot \left|\mathrm{H}\left(q - \frac{k}{\Delta x}\right)\right|^2 \cdot \mathrm{rect}(\mathrm{q}\,\Delta x - \mathrm{k}) \qquad (3)$$

In Eq. (3), $\left(q - \frac{k}{\Delta x}\right)^2$ is the contribution of the Ram-Lak filter, $\left(sinc^2(\pi q \Delta x)\right)^2$ the low-pass filtering introduced by the linear interpolations and $\mathrm{H}\left(q - \frac{k}{\Delta x}\right)$ is the apodization function which can be employed to reduce the high frequency components of the noise. The expressions for some function $H(q)$ commonly employed in FBP are listed in Table A1.

Filter	$H(q)$
Shepp-Logan	$sinc(\pi q \Delta x)$
Hamming	$0.54 + 0.46\, cos(2\pi q \Delta x)$
Hann	$0.5 + 0.5\, cos(2\pi q \Delta x)$

Table A1: List of some apodization functions commonly employed in FBP algorithm

The noise variance for each reconstructed point derives from Eq. (1) and, in polar coordinates, is [2]

$$\sigma^2 = \int_0^{2\pi} d\theta \int_0^{+\infty} NPS(q)\, q\, dq = \frac{2\pi^2 \Delta x}{m\langle n\rangle} \cdot \int_0^{+\infty} |W(q)|^2\, dq \qquad (4)$$

Moreover, the function

$$rect(\mathrm{x}) = \begin{cases} 1, |x| < 1/2 \\ 0, \text{ otherwise} \end{cases} \qquad (5)$$

allows to rewrite Eq. (3) as a piecewise function

$$|W(q)|^2 = \begin{cases} (q)^2 \cdot |\mathrm{H}(q)|^2 \cdot \left(sinc^2(\pi q \Delta x)\right)^2, & 0 < \mathrm{q} < \frac{1}{2\Delta x}, \quad k = 0 \\ \left(q - \frac{k}{\Delta x}\right)^2 \cdot \left|\mathrm{H}\left(q - \frac{k}{\Delta x}\right)\right|^2 \cdot \left(sinc^2(\pi q \Delta x)\right)^2, & \left(\frac{2k-1}{2\Delta x}\right) < \mathrm{q} < \left(\frac{2k+1}{2\Delta x}\right), \mathrm{k} \neq 0 \end{cases} \qquad (6)$$

By combining Eq. (4) and Eq. (6) we obtain

$$\sigma^2 = \frac{2\pi^2 \Delta x}{m\langle n\rangle}\left(\int_0^{1/2\Delta x} |W(q)|^2\, dq + \sum_k \int_{(2k-1)/2\Delta x}^{(2k+1)/2\Delta x} |W(q)|^2\, dq\right) \equiv \frac{\beta}{m\langle n\rangle} \qquad (7)$$

where β has the dimension of a squared spatial frequency.

The Nyquist frequency (q_{Ny}) of a CT image sampled with a voxel of linear size Δx, is $q_{Ny} = 1/2\Delta x$. In the backprojection process, the frequency components of the projection data which exceed q_{Ny} are folded back into a region below q_{Ny} due to the discrete sampling. In Eq. (7) this means that the integrals in the summation (of $|W(q)|^2$ for $q > q_{Ny}$) are the contributions of the aliasing effect to the global noise as shown in Figure A1.

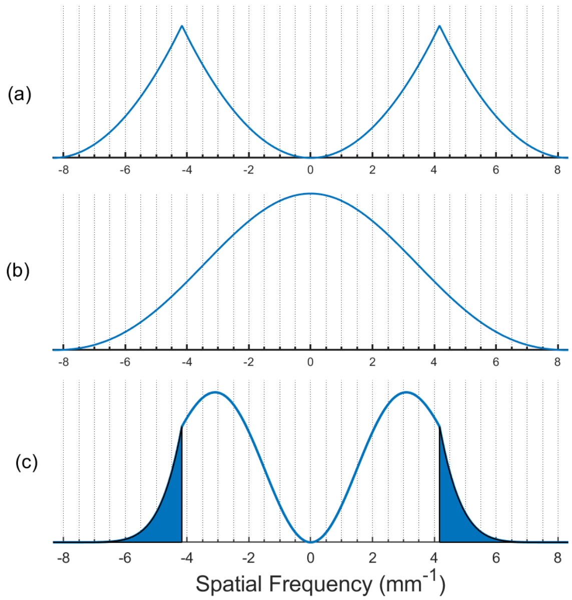

Figure A1: $\Delta x = 0.120\ mm$ (a) frequency response of the square of Ram-Lak filter; (b) frequency response of the square of the linear interpolation kernel, i.e. $\left(sinc^2(\pi q \Delta x)\right)^2$; (c) spectral response of the reconstruction algorithm ($|W(q)|^2$, Eq. (3)). The shaded area is the noise which is folded back (aliasing).

The integrals in Eq. (7) can be evaluated numerically. We used Matlab for numerical integrations and, for a pixel of size $\Delta x = 0.12\ mm$ and a Ram-Lak filter, we obtained

$$\sigma^2_{Ram-Lak} = \frac{\beta_{Ram-Lak}}{m\langle n\rangle} \approx \frac{2\pi^2 \Delta x}{m\langle n\rangle}(9.448 + \mathbf{1.737}) \approx \frac{26.49}{m\langle n\rangle}\ mm^{-2} \qquad (8)$$

In Eq. (8) the contribution of Aliasing to the noise is in bold. The results for Eq. (7) when using the apodization windows listed in Table A1 are reported in Table A2.

Filter	σ^2	$\beta\ (mm^{-2})$
Ram-Lak	$\frac{2\pi^2 \Delta x}{m\langle n\rangle}(9.448 + \mathbf{1.737}) \approx \frac{26.49}{m\langle n\rangle}\ mm^{-2}$	$\beta_{Ram-Lak} = 26.49$
Shepp-Logan	$\frac{2\pi^2 \Delta x}{m\langle n\rangle}(6.457 + \mathbf{0.8822}) \approx \frac{17.38}{m\langle n\rangle}\ mm^{-2}$	$\beta_{Shepp-Logan} = 17.38$
Hamming	$\frac{2\pi^2 \Delta x}{m\langle n\rangle}(1.8169 + \mathbf{0.0457}) \approx \frac{4.411}{m\langle n\rangle}\ mm^{-2}$	$\beta_{Hamming} = 4.411$
Hann	$\frac{2\pi^2 \Delta x}{m\langle n\rangle}(1.5679 + \mathbf{0.0224}) \approx \frac{3.714}{m\langle n\rangle}\ mm^{-2}$	$\beta_{Hann} = 3.714$

Table A2: σ^2 for different FBP filters

The noise variance calculated in Eq. (7) is function of the mean number of registered X-ray quanta $\langle n \rangle$. According to the Lambert-Beer law, considering a CT scan with a monochromatic parallel beam of energy E, $\langle n \rangle$ is function of the number of incident quanta to the sample $\langle n \rangle_0$, the geometry of the sample and the distribution of the linear attenuation coefficients $\mu_{Sample}(x, y, E)$ of which the sample is made. For a homogeneous circular sample of radius R, the transmitted X-ray quanta $\langle n \rangle$ passing from the center is:

$$\langle n \rangle = \langle n \rangle_0 \cdot e^{-2\int_0^R \mu_{Sample}\, dR} = \langle n \rangle_0 \cdot e^{-2\mu_{Sample} \cdot R} \qquad (9)$$

Indicating the total number of photons per pixel impinging on the phantom as $N_{ph} = m\langle n \rangle_0$ and by combining (7) and (9), the variance $\sigma^2(0,0)$ at the center of the CT reconstruction with FBP is:

$$\sigma^2(0,0) = \frac{\beta}{N_{ph} \cdot e^{-2\mu_{Sample} \cdot R}} \qquad (10)$$

We underline that, if the sample is placed at a some distance D from the detector and the space is filled with air, the Eq. (10) has to be corrected for the attenuation of the air:

$$\sigma^2_{corr}(0,0) = \frac{\beta}{N_{ph} \cdot e^{-2\mu_{Sample} \cdot R}} \cdot \frac{1}{e^{-\mu_{air} \cdot D}} \qquad (11)$$

where μ_{air} is the linear attenuation coefficient of the air.

References

1. Cunningham I. Applied Linear-Systems Theory. In: *Van Metter R. L., Beutel J., Kundel H. L. Handbook of Medical Imaging* Vol. 1. Bellingham:Press SPIE; 2000:79-162.

2. Riederer, S. J., Pelc, N. J. & Chesler, D. A., The noise power spectrum in computed X-ray tomography. *Phys. Med. Biol.* **23** (3), 446, https://doi.org/10.1088/0031-9155/23/3/008 (1978).

3. Hanson, K. M., Detectability in computed tomographic images. *Med. Phys.* **6** (5), 441-451, https://doi.org/10.1118/1.594534 (1979).

4. Wagner, R. F., Brown, D. G. & Pastel, M. S., Application of information theory to the assessment of computed tomography. *Med. Phys.* **6** (2), 83-94, https://doi.org/10.1118/1.594559 (1979).

5. Faulkner, K. & Moores, B. M., Noise and contrast detection in computed tomography images. *Phys. Med. Biol.* **29** (4), 329, https://doi.org/10.1088/0031-9155/29/4/003 (1984).

6. Kijewski, M. F. & Judy, P. F., The noise power spectrum of CT images. *Phys. Med. Biol* **32** (5), 565, https://doi.org/10.1088/0031-9155/32/5/003 (1987).
